# Taxonomy and Distribution of the Genus *Sellaphora* Mereschowsky (Bacillariophyceae: Sellaphoraceae) in Aquatic Ecosystems of Mongolia

**DOI:** 10.3390/plants12203611

**Published:** 2023-10-18

**Authors:** Anton Glushchenko, Sergei Genkal, Irina Kuznetsova, Maxim Kulikovskiy

**Affiliations:** К.А. Timiryazev Institute of Plant Physiology RAS, IPP RAS, 35 Botanicheskaya Street, 127276 Moscow, Russiamax-kulikovsky@yandex.ru (M.K.)

**Keywords:** Bacillariophyceae, new species, new records, *Sellaphora*, systematics, taxonomy, morphology, Mongolia

## Abstract

During our investigation, 28 species belonging to the genus *Sellaphora* Mereschowsky were found in different types of water bodies and streams in Mongolia. Of these, 14 species have already been described and/or shown earlier in the flora of Mongolia, and we found 14 species for the first time. One new taxonomic combination has been proposed: *Sellaphora glomus* (J.R. Carter and A.E. Bailey-Watts) Glushchenko and Kulikovskiy comb. nov. One species is proposed for description as new to science: *Sellaphora dorofeyukae* Glushchenko and Kulikovskiy sp. nov. All species were illustrated with original light micrographs, and a number of species were also illustrated with scanning electronic photographs. The distribution of the identified species within Mongolia and the world is discussed.

## 1. Introduction

The genus *Sellaphora* was described by K. Mereschkowsky in 1902 г. [1]. This genus includes more than 200 species [2]. This is the largest genus in the family Sellaphoraceae. This family also includes following genera *Buryatia* Kulikovskiy, Lange-Bertalot and Metzeltin; *Caponea* Podzorski; *Dimidiata* Hajos; *Diprora* Main; *Eolimna* Lange-Bertalot and Schiller; *Fallacia* Stickle and D.G. Mann; *Lacuneolimna* Tudesque, Le Cohu, and Lange-Bertalot; *Okhapkinia* Glushchenko, Kulikovskiy and Kociolek, *Rossia* Voigt and *Chamaepinnularia* [2,3,4,5,6]. Species of the genus *Sellaphora* have a worldwide distribution, inhabiting different types of water bodies and soils [3]. Species new to science are constantly being described, including those from Asia [7,8,9,10,11,12].

The few revisions of the genus *Sellaphora* were prepared by different authors [13,14,15]; however, these revisions had no floristic orientation and concerned the study of taxonomic histories of different species. Diatoms from different ecosystems in Mongolia have been studied for more than 100 years [16]. According to the latest data in the literature, at least 30 species of *Sellaphora* are known from Mongolia [17], which we list in Table 1, of which 13 were first described directly from aquatic ecosystems or their bottom sediments in this country [18].

This is a rather small number of species for such a vast floristically interesting territory. Thus, there is a need for a new revision of the genus *Sellaphora* from the aquatic ecosystems of Mongolia.

A complex relief and climatic conditions determine the size and variety of Mongolian aquatic ecosystems. Mongolian water resources are limited and unevenly common. The lakes and the rivers of Mongolia belong to the water collection pools of the Arctic and Pacific Oceans, as well as to the Central Asian basin [17].

The aim of this publication is investigation of the diversity, morphology, taxonomy, and distribution of *Sellaphora* species from Mongolia with the description of a new species.

## 2. Results

Of the 30 species previously known in the flora of Mongolia, 16 have been described from this region (see Table 1).

During this investigation, 28 species belonging to the genus *Sellaphora* were found.

Of them:

- Fourteen species—previously known in the flora of Mongolia according to literary data (including 11 described directly from the country’s aquatic ecosystems);
- Thirteen species—known from other regions and shown for the first time in the flora of Mongolia;
- One species—new to science.

Main morphometric data for the investigated species and a list of them are given below.

**Figure 1:**
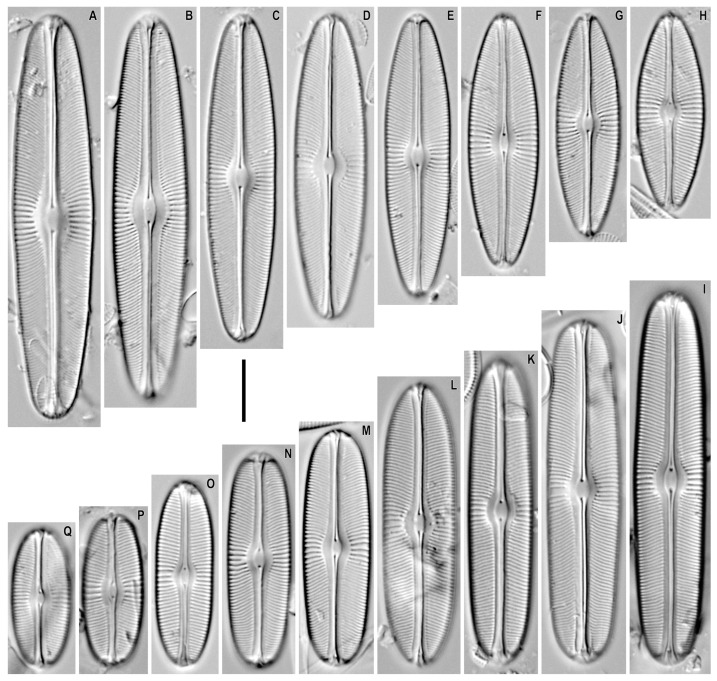
*Sellaphora* spp. Light microscopy, differential interference contrast, size diminution series. (**A**–**H**). *S. pseudobacillum*. Slide no. 02605 (**A**–**H**). (**I**–**Q**). *S. mongolocollegarum.* Slide no. 02475 (**M**), 02622 (**I**), 02642 (**P**), 02687 (**L**), 03053 (**J**), 03097, (**K**,**O**) 03149, (**N**), 03151 (**Q**). Scale bar = 10 μm.

*Sellaphora pseudobacillum* (Grunow) Lange-Bertalot and Metzeltin in Metzeltin et al. 2009 (Figure 1A–H).

**Remarks.** The specimens in our samples were 31.5–64.5 μm long, 10.2–13.0 μm wide, and had 18–19 striae in 10 μm at the central part and 20–21 striae in 10 μm near the ends.

This species was found on slide no. 02605.

*Sellaphora mongolocollegarum* Metzeltin and Lange-Bertalot in Metzeltin et al. 2009 (Figure 1I–Q).

**Remarks.** The specimens in our samples were 22.0–59.4 μm long, 9.1–11.6 μm wide, and had 19–21 striae in 10 μm at the central part and 22–24 striae in 10 μm near the ends.

This species was found on slides no. 02475, 02622, 02642, 02687, 03053, 03097, 03149, and 03151.

**Figure 2:**
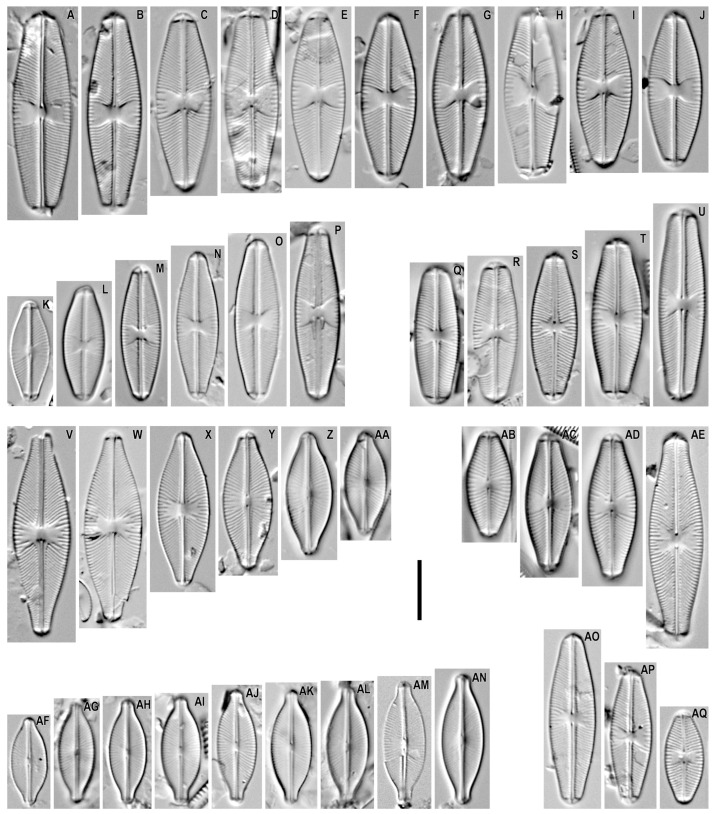
*Sellaphora* spp. Light microscopy, differential interference contrast, size diminution series. (**A**–**J**). *S. perobesa*. (**K**–**P**). *S. ellipticolanceolata*. (**Q**–**U**). *S. obesa*. (**V**–**AA**). *S. permutata*. (**AB**–**AE**). *S. auldreekie*. (**AF**–**AN**). *S. mutata*. (**AO**–**AQ**). *S. lanceolata*. Slide no. 02457 (**Q**), 02463 (**E**,**F**,**G**,**I**,**J**,**N**,**W**,**X**), 02474 (**V**), 02605 (**S**,**AE**,**AO**,**AQ**), 02611 (**U**), 02642 (**D**,**H**,**R**,**AM**), 02963 (**B**,**C**), 02984 (**A**), 03097 (**O**), 03101 (**AP**), 03105 (**L**,**AJ**,**AN**), 03121 (**K**,**M**), 03151 (**Z**,**AA**,**AB**–**AD**), 03163 (**Y**,**AF**–**AI**,**AK**,**AL**), 03169 (**P**), 03183 (**T**). Scale bar = 10 μm.

*Sellaphora perobesa* Metzeltin, Lange-Bertalot and Nergui 2009 (Figure 2A–J).

**Remarks.** The specimens in our samples were 26.2–33.8 μm long, 9.0–9.9 μm wide, and had 20–22 striae in 10 μm.

This species was found on slides no. 02463, 02642, and 02984.

*Sellaphora ellipticolanceolata* Metzeltin, Lange-Bertalot and Nergui 2009 (Figure 2K–P).

**Remarks.** The specimens in our samples were 16.9–29.0 μm long, 6.6–7.5 μm wide, and had 21–22 striae in 10 μm.

This species was found on slides no. 02463, 03097, 03105, 03121, and 03169.

*Sellaphora obesa* D.G. Mann and M.M. Bayer in Mann et al. 2004 (Figure 2Q–U).

**Remarks.** The specimens in our samples were 23.2–32.2 μm long, 7.3–8.5 μm wide, and had 21–22 striae in 10 μm.

This species was found on slides no. 02457, 02605, 02611, 02642, and 03183.

*Sellaphora permutata* Metzeltin, Lange-Bertalot and Nergui 2009 (Figure 2V–AA).

**Remarks.** The specimens in our samples were 17.2–34.1 μm long, 7.1–9.7 μm wide, and had 20–25 striae in 10 μm.

This species was found on slides no. 02463, 02474, 03151, and 03163.

*Sellaphora auldreekie* D.G. Mann and S.M. McDonald in Mann et al. 2004 (Figure 2AB–AE).

**Remarks.** The specimens in our samples were 17.8–35.0 μm long, 7.9–9.3 μm wide, and had 18–22 striae in 10 μm.

This species was found on slides no. 02605 and 03151.

*Sellaphora mutata* (Krasske) Lange-Bertalot in Lange-Bertalot et al. 1996 (Figure 2AF–AN).

**Remarks.** The specimens in our samples were 14.8–21.9 μm long, 6.1–7.1 μm wide, and had 22–24 striae in 10 μm.

This species was found on slides no. 02642, 03105, 03121, and 03163.

*Sellaphora lanceolata* D.G. Mann and S. Droop in Mann et al. 2004 (Figure 2AO–AQ).

**Remarks.** The specimens in our samples were 15.6–28.5 μm long, 6.6–7.8 μm wide, and had 23–24 striae in 10 μm.

This species was found on slides no. 02605 and 03101.

**Figure 3:**
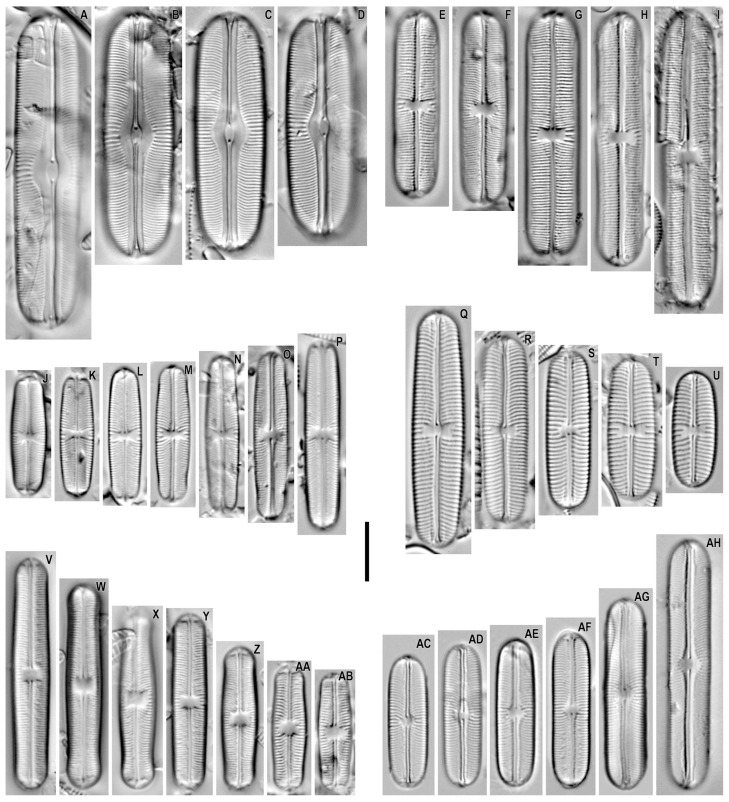
*Sellaphora* spp. Light microscopy, differential interference contrast, size diminution series. (**A**–**D**). *S. boltziana*. (**E**–**I**). *S. khangalis*. (**J**–**P**). *S. pseudopupula*. (**Q**–**U**). *S. perlaevissima*. (**V**–**AB**). *S. kusberi*. (**AC**–**AH**). *S. pelagonica*. Slide no. 02444 (**Q**), 02447 (**T**), 02605 (**I**,**AH**), 02611 (**A**,**C**,**Z**), 02620 (**E**–**G**,**H**,**W**,**Y**), 02636 (**U**), 02642 (**B**,**D**,**R**,**AD**), 02683 (**AC**), 02684 (**AG**), 03005 (**AE**), 03015 (**V**), 03097 (**S**), 03150 (**X**,**AA**,**AB**), 03105 (**J**), 03017 (**AC**), 03121 (**L**), 03123 (**M**,**O**), 03163 (**K**,**N**) 03169 (**P**,**AF**). Scale bar = 10 μm.

*Sellaphora boltziana* Metzeltin, Lange-Bertalot and Nergui 2009 (Figure 3A–D).

**Remarks.** The specimens in our samples were 37.7–53.3 μm long, 11.2–11.8 μm wide, and had 20–22 striae in 10 μm.

This species was found on slides no. 02611 and 02642.

*Sellaphora khangalis* Metzeltin and Lange-Bertalot in Metzeltin et al. 2009 (Figure 3E–I).

**Remarks.** The specimens in our samples were 31.6–50.1 μm long, 7.3–9.0 μm wide, and had 19–22 striae in 10 μm.

This species was found on slides no. 02605 and 02620.

*Sellaphora pseudopupula* (Krasske) Lange-Bertalot in Lange-Bertalot et al. 1996 (Figure 3J–P).

**Remarks.** The specimens in our samples were 20.2–31.9 μm long, 5.5–5.8 μm wide, and had 22–23 striae in 10 μm.

This species was found on slides no. 03105, 03121, 03123, 03163, and 03169.

*Sellaphora perlaevissima* Metzeltin, Lange-Bertalot and Nergui 2009 (Figure 3Q–U and Figure 5E,F).

**Remarks.** The specimens in our samples were 19.4–39.4 μm long, 7.4–8.7 μm wide, and had 15–16 striae in 10 μm.

This species was found on slides no. 02444, 02447, 02636, 02642, and 03097.

Note: 

*Sellaphora kusberi* Metzeltin, Lange-Bertalot and Nergui 2009 (Figure 3V–AB).

**Remarks.** The specimens in our samples were 19.7–38.6 μm long, 5.6–6.3 μm wide, and had 22–23 striae in 10 μm.

This species was found on slides no. 02447, 02611, 02620, 03015, and 03150.

*Sellaphora pelagonica* Kochoska, Zaova, Videska and Levkov in Kochoska et al. 2021 (Figure 3AC–AH).

**Remarks.** The specimens in our samples were 22.2–41.6 μm long, 6.1–7.2 μm wide, and had 23–25 striae in 10 μm.

This species was found on slides no. 02605, 02642, 02684, 03005, and 03169.

**Figure 4:**
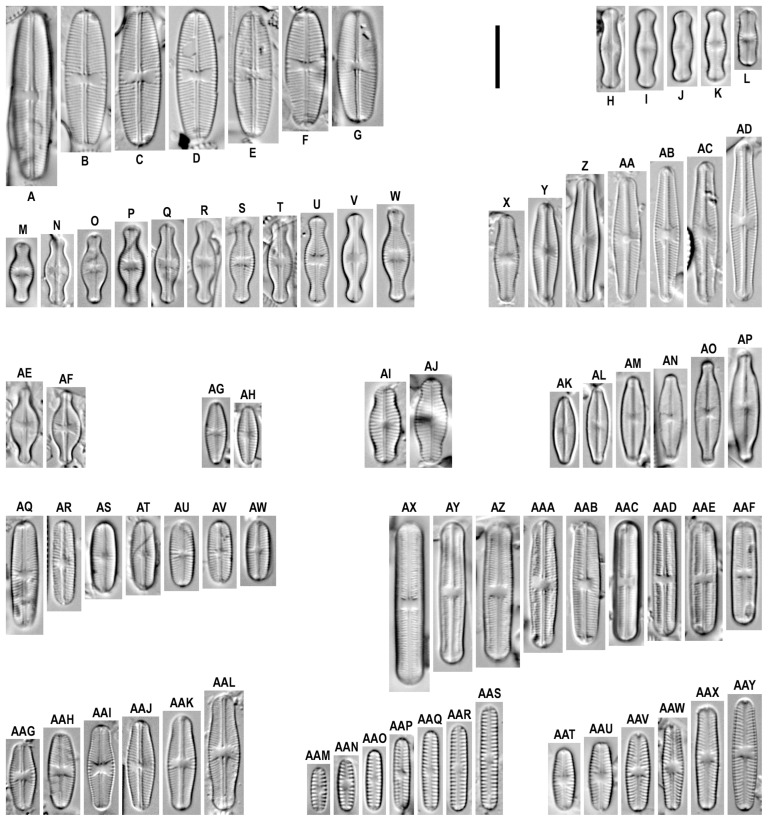
*Sellaphora* spp. Light microscopy, differential interference contrast, size diminution series. (**A**–**G**). *S. bisexualis*. (**H**–**L**). *S. medioconvexa*. (**M**–**W**). *S. digna*. (**X**–**AD**). *S. disjuncta*. (**AE**,**AF**). *S. glomus* comb. nov. (**AG**,**AH**). *S. atomoides*. (**AI**,**AJ**). *S. pseudoventralis*. (**AK**–**AP**). *S. cosmopolitana*. (**AQ**–**AW**). *S. stroemii*. (**AX**–**AAF**). *S. intermissa*. (**AAG**–**AAL**). *S. multiconfusa*. (**AAM**–**AAS**). *S. vekhovii*. (**AAT**–**AAY**). *S. seminulum*. Slide no. 02431 (**I**,**K**,**AI**,**AAK)**, 02440 (**M**,**O**), 02447 (**B**,**C**,**H**,**L**,**M**,**N**,**Q**,**AJ**,**AAJ**,**AAL**,**AAM**), 02457 (**J**,**AC**,**AAN**), 02458 (**AK**–**AP**,**AAO**), 02478 (**AAY**), 02601 (**AAX**), 02620 (**AB**,**AX**,**AAB**,**AAS**), 02622 (**A**), 02636 (**F**), 02642 (**D**,**E**,**G**,**P**,**R**–**Z**,**AD**–**AF**,**AH**,**AAA**,**AAD**,**AAG**–**AAI**,**AY**), 02675 (**AA**), 03015 (**AAQ**), 03016 (**AAR**) 03123 (**AAT**–**AAW**), 03027 (**AAP**), 03030 (**AG**), 03183 (**AQ**–**AW**), 03163 (**AZ**), 02696 (**AAC**), 03167 (**AAE**,**AAF**). Scale bar = 10 μm.

*Sellaphora bisexualis* D.G. Mann and D.G. Mann et Evans in Mann et al. 2009 (Figure 4A–G).

**Remarks.** The specimens in our samples were 18.1–27.0 μm long, 6.1–6.9 μm wide, and had 20–23 striae in 10 μm.

This species was found on slides no. 02447, 02622, 02636, and 02642.

**Figure 5:**
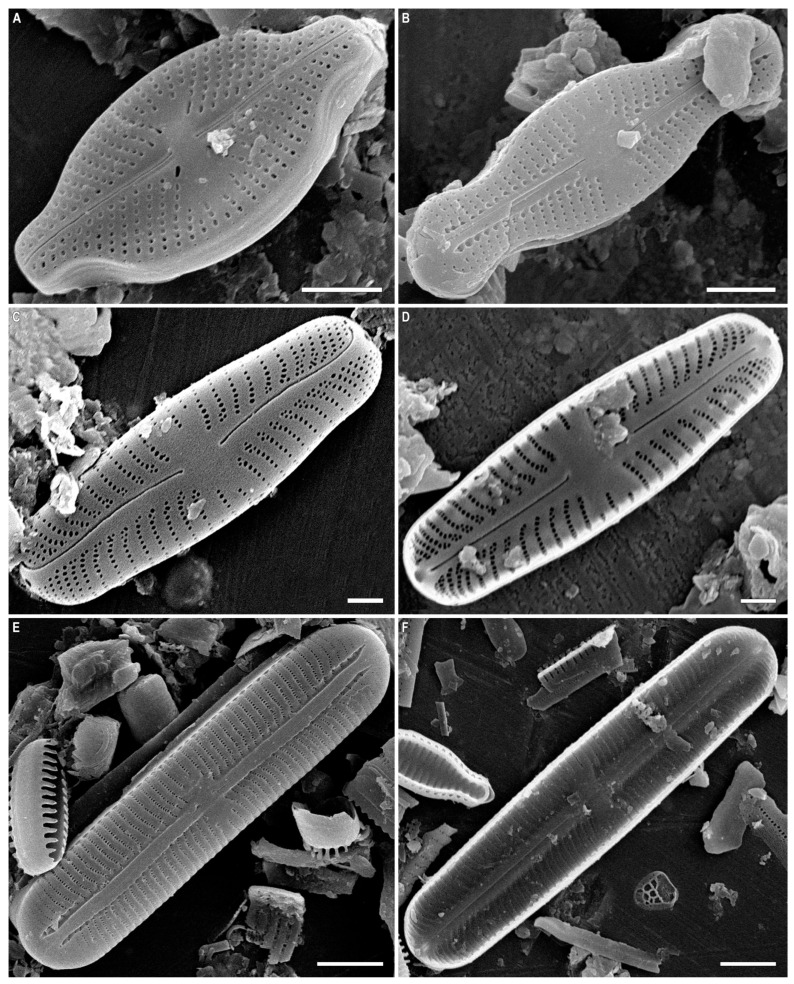
*Sellaphora* spp. Scanning electron microscopy, external views (**A**–**C**,**E**), internal views (**D**,**F**). (**A**). *Sellaphora glomus* comb. nov. (**B**). *Sellaphora medioconvexa*. (**C**,**D**). *Sellaphora seminulum*. (**E**,**F**). *Sellaphora perlaevissima*. (**A**,**B**,**E**,**F**) Sample no. Mn289.1 (corresponds to the slide no. 02642). (**C**,**D**) Sample no. Mn195 (corresponds to the slide no. 03123). Scale bar (**E**,**F**) = 5 μm; (**A**,**B**) = 2 μm; (**C**,**D**) = 1 μm.

*Sellaphora medioconvexa* (Hustedt) Wetzel in Wetzel et al. 2015 (Figure 4H–L and Figure 5B).

**Remarks.** The specimens in our samples were 8.8–12.7 μm long, 3.1–3.6 μm wide, and had 34 striae in 10 μm.

This species was found on slides no. 02431, 02447, and 02457.

*Sellaphora digna* (Hustedt) Wetzel, Ector, Van de Vijver, Compère and D.G. Mann 2015 (Figure 4M–W).

**Remarks.** The specimens in our samples were 9.3–14.7 μm long, 3.8–4.4 μm wide, and had 33–36 striae in 10 μm.

This species was found on slides no. 02440, 02447, and 02642.

*Sellaphora disjuncta* (Hustedt) D.G. Mann 1989 (Figure 4X–AD).

**Remarks.** The specimens in our samples were 14.0–25.4 μm long, 4.0–4.5 μm wide, and had 24–25 striae in 10 μm.

This species was found on slides no. 02457, 02620, 02642, and 02675.

*Sellaphora atomoides* (Grunow) Wetzel et Van de Vijver in Wetzel et al. 2015 (Figure 4AG,AH and Figure 5C,D).

**Remarks.** The specimens in our samples were 9.1–9.8 μm long, 3.2–3.5 μm wide, and had 30–32 striae in 10 μm.

This species was found on slides no. 02642 and 03030.

*Sellaphora pseudoventralis* (Hustedt) Chudaev and Gololobova 2015 (Figure 4AI,AJ).

**Remarks.** The specimens in our samples were 12.3–13.6 μm long, 5.1–5.3 μm wide, and had 20–21 striae in 10 μm.

This species was found on slides no. 02431 and 02447.

*Sellaphora cosmopolitana* (Lange-Bertalot) Wetzel and Ector in Wetzel et al. 2015 (Figure 4AK–AP).

**Remarks.** The specimens in our samples were 10.7–12.8 μm long, 3.5–4.3 μm wide. Striae not resolved in LM.

This species was found on slides no. 02458.

*Sellaphora stroemii* (Hustedt) H. Kobayasi in Mayama et al. 2002 (Figure 4AQ–AW).

**Remarks.** The specimens in our samples were 17.1–9.6 μm long, 3.8–4.5 μm wide, and had 24–25 striae in 10 μm.

This species was found on slide no. 03183.

*Sellaphora intermissa* Metzeltin, Lange-Bertalot et Nergui 2009 (Figure 4AX–AAF).

**Remarks.** The specimens in our samples were 16.3–26.1 μm long, 3.8–4.6 μm wide, and had 24–27 striae in 10 μm.

This species was found on slides no. 02463, 02620, 02642, 02696, 03163, and 03167.

*Sellaphora multiconfusa* (Lange-Bertalot) Wetzel, Ector, Van de Vijver, Compère et D.G. Mann 2015 (Figure 4AAG–AAL).

**Remarks.** The specimens in our samples were 10.7–18.3μm long, 4.2–5.0 μm wide, and had 27–28 striae in 10 μm.

This species was found on slides no. 02431, 02447, and 02642.

*Sellaphora vekhovii* (Lange-Bertalot et Genkal) Wetzel et D.G. Mann in Wetzel et al. 2015 (Figure 4AAM–AAS).

**Remarks.** The specimens in our samples were 7.0–16.6 μm long, 2.4–2.7 μm wide, and had 16–17 striae in 10 μm.

This species was found on slides no. 02447, 02457, 02458, 02620, 03015, 03016, and 03027.

*Sellaphora seminulum* (Grunow) D.G. Mann 1989 (Figure 4AAT–AAY and Figure 5C,D).

**Remarks.** The specimens in our samples were 9.9–18.0 μm long, 3.6–3.7 μm wide, and had 25 striae in 10 μm.

This species was found on slides no. 02478, 02621, 03123.

**New combination:**

*Sellaphora glomus* (J.R. Carter and A.E. Bailey-Watts) Glushchenko and Kulikovskiy comb. nov. (Figure 4AE,AF and Figure 5A).

**Basionym:***Navicula glomus* J.R. Carter and A.E. Bailey-Watts 1981. A taxonomic study of diatoms from standing freshwaters in Shetland. Nova Hedwigia. Vol. 33. Issue 3/4. P. 578–579. Pl. 13. Figure 17.

**Remarks.** The specimens in our sample were 11.9–12.1 μm long, 4.5–4.6 μm wide, and had 30 striae in 10 μm.

This species was found on slide no. 02642.

**Figure 6:**
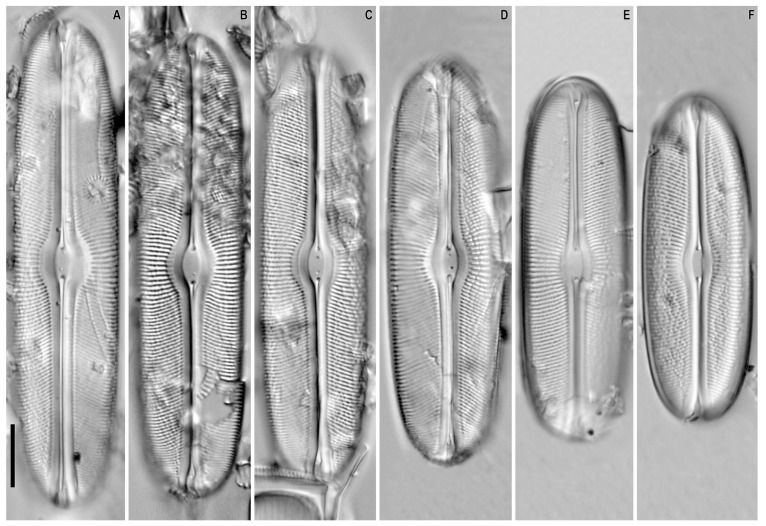
*Sellaphora dorofeyukae* Glushchenko and Kulikovskiy sp. nov. Light microscopy, differential interference contrast, size diminution series. (**A**). Holotype. Davaa Lake. Slide no. 02437 (**A**,**D**), 02451 (**B**), 03101 (**C**,**E**,**F**). Scale bar = 10 μm.

**Figure 7:**
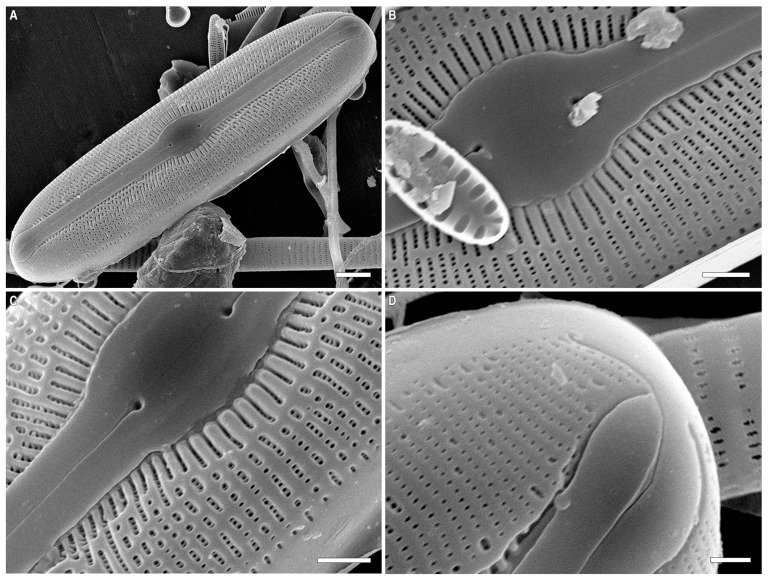
(**A**–**D**). *Sellaphora dorofeyukae* Glushchenko and Kulikovskiy sp. nov. Scanning electron microscopy, external views. (**A**). The whole valve. (**B**,**C**). Central area. (**D**). Valve end. Scale bar (**A**) = 5 μm; (**B**,**C**) = 2 μm; (**D**) = 1 μm.

**Figure 8:**
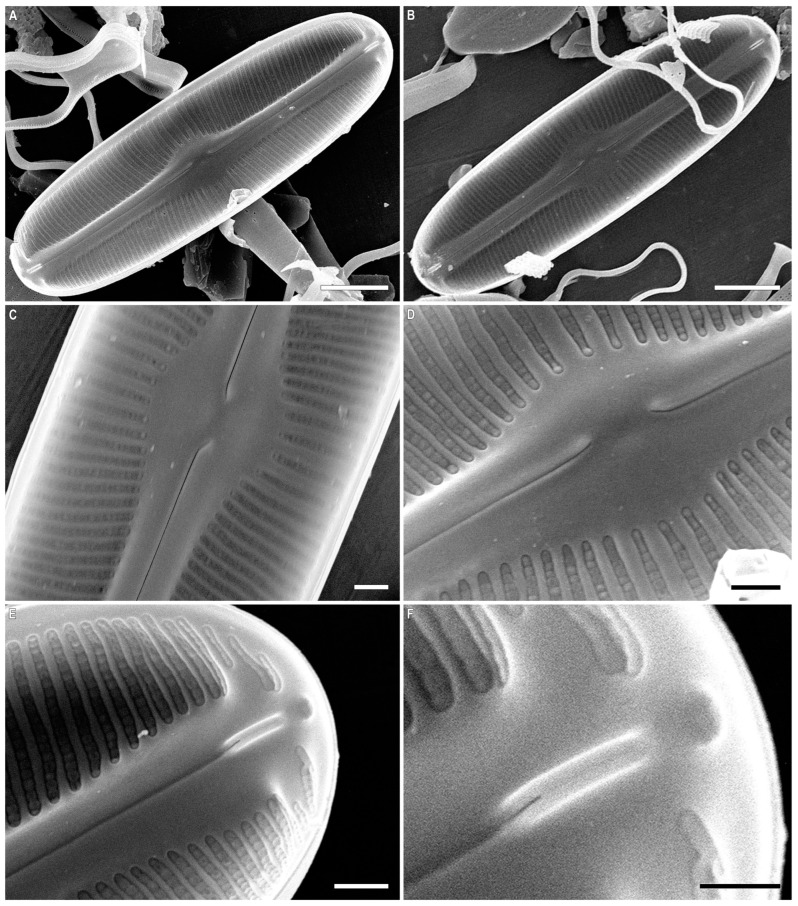
(**A**–**F**). *Sellaphora dorofeyukae* Glushchenko and Kulikovskiy sp. nov. Scanning electron microscopy, internal views. (**A**,**B**).The whole valve. (**C**,**D**). Central area. (**E**,**F**). Valve end. Scale bar (**A**,**B**) = 5 μm; (**C**–**E**) = 1 μm; (**F**) = 0.5 μm.

New species:

*Sellaphora dorofeyukae* Glushchenko and Kulikovskiy sp. nov. (Figure 6A–F, Figure 7A–D and Figure 8A–F).

**Holotype.** Collection of Maxim Kulikovskiy at the Herbarium of the Institute of Plant Physiology Russian Academy of Science, Moscow, Russia, holotype here designated, slide No. 02437 (Figure 6A).

**Type locality**. Mongolia, Lake Davaa, periphyton, 48°10.803′ N; 98°46.107′ E. Collected by M.S. Kulikovskiy, 8 July 2015, pH = 8.1, conductivity = 20 µS cm^–1^, t = 13 °C.

**Etymology**. Species dedicated to well-known algologist and investigator of Mongolian algae Dr. Nadezda Ivanovna Dorofeyuk.

**Distribution.** Known from the Davaa Lake.

**Description.** LM (Figure 6A–F). Valves linear with parallel margins and broadly rounded ends.

Length 52.1–77.8 µm, breadth 16.6–18.4 µm. Axial area is narrow. Central area apically broad-elliptical, extended to 1/3–1/4 of the valve breadth. Central raphe ends extended drop-like, expanded pores. At the central nodule near the central raphe ends are sometimes present one or two small structures, similar to round (Figure 6A–D). Distal raphe ends deflected onto the valve mantle. Striae subparallel near the central part, then become radiate, 16–19 in 10 µm.

SEM, external view (Figure 7A–D). The valve is silicified (Figure 7A). The outer silica layer has perforations (further lacunae, by Metzeltin and Lange-Bertalot), through which individual areolae located on the basal layer are visible (Figure 7B,C). The lacunae are more or less transversely elongated and vary in depth. The lacunae adjacent to the raphe-sternum closer to the central area are deeper and transversely elongated and may be open from the side adjacent to the raphe-sternum. Areolae in lacunae adjacent to raphe-sternum are smaller (Figure 7B). The lacunae located in the transapical part of the valve are predominantly shorter. The number of areolae in lacunae ranges from 2 to 10. Additionally, in the central part of the valve, there are small single rounded areolae without visible lacunae. Closer to the ends of the valves, the lacunar device is replaced by the usual small rounded areolae, especially closer to the valve margins.

The valve margins are smooth and devoid of areolae (Figure 7A,D). Two distinct longitudinal depressions are present on either side of the raphe-sternum. Encompassing the complex of raphe-sternum, flaps of the conopeum and parallel apical depressions are straight (not undulating in parts), ca. 3 μm broad (Figure 7A–C).

The density of areolae in lacunae is 50 in 10 µm in the central part of the valve, up to 70 in 10 μm in lacunae located near the central area. The density of areolae at the valve ends is 40–45 in 10 μm. The distal raphe end is bent to the valve margin, located parallel and fairly close to one of the stria (Figure 7D).

SEM, internal view (Figure 8A–F). The raphe is straight, located on a slightly raised sternum (Figure 8A–E). The central raphe ends are bent to one side. Distal raphe ends terminate in narrow helictoglossae. Helictoglossae are located near the apical pits (Figure 8F). The polar bar is well expressed (Figure 8A,E). Several rows of short striae are located on the polar bar (Figure 8E). Uniseriate striae consist of areolae covered with rounded or rectangular hymenes (Figure 8D,E).

## 3. Discussion

### 3.1. Species Previously Described or/and Shown in Mongolia

*Sellaphora pseudobacillum* has previously been shown in the plankton and benthos of lakes Khövsgöl and Hangal, as well as for the benthos of the Changa and Tuul rivers [17]. It was also found by us in Lake Khövsgöl. The species has a Holarctic distribution, apparently often identified as *S. bacillum* s.l. On the whole, our finding of *S. pseudobacillum* fits into the type description of the species.

*Sellaphora mongolocollegarum* was previously shown in the benthos of the Ynegtiin and Shurguitm Jargalant rivers and Lake Hangal [17]. We have shown new localities of this species from lakes Bayan, Khövsgöl (and the nameless reservoir adjacent to it separated by a sandy spit), Holbo, Ögii, as well as in the Suman River. In general, our finding of *S. mongolocollegarum* fits into the morphology of the original description of this species.

*Sellaphora perobesa* was previously shown only in the benthos of Lake Hangal [17]. We found it in Lake Bayan and in an unnamed reservoir separated by a sandy spit from Lake Khövsgöl. Our material differs from the typical material, with a somewhat higher density of striae in 10 µm (18–21 in the type material versus 20–22 in our material). The species is known only from Mongolia.

*Sellaphora ellipticolanceolata* was previously recorded in the sediments of lakes Hangal and Buyr, as well as in the benthos and epiphyton of small ponds of Buyr Lake catchment area Hangal [17]. We illustrate new localities of this species from lakes Bayan, Terkhiin Tsagaan, as well as the rivers Suman and Terkhiin. We found valves smaller than indicated in the original description (length from 16.9 µm, width from 6.6 µm in our sample versus 21 µm in length and 7 µm in width for the type material). The species is known from Mongolia, but some specimens of *Sellaphora* from Bear Island can be interpreted as *S. ellipticolanceolata* ([19], Figure 7, pp. 10–12). Thus, the distribution of the species requires clarification.

*Sellaphora permutata* was found in the benthos of the Herlen River [17]. We show new locations at lakes Bayan, Ögii, Zavagitn. Our material fits into the original description of this species. The species is known from Mongolia.

*Sellaphora auldreekie* was previously shown in the sediments of Great Lakes Depression (sine loco) lakes [17]. We demonstrate new findings of this species outside this basin from lakes Khövsgöl and Ögii. Our studied population differs from the type material by a somewhat larger width of valves (6.6–8.0 µm in the original description versus 7.9–9.3 µm in our material). The species was described from Great Britain [13] but, apparently, is more widely distributed and can be identified as *S. pupula* s.l.

*Sellaphora mutata* was previously found in the plankton of the Hag and Buyr lakes, as well as in the plankton of the Urd Tamir River [17]. We have shown new localities of this species in the lakes Terkhiin Tsagaan and Zavagitn Nur as well as in an unnamed reservoir separated from Lake Khövsgöl by a sandy spit. The species has a Holarctic distribution.

*Sellaphora boltziana* was found in the benthos of Lake Hoh. The new location was an unnamed reservoir separated by a sandy spit from Lake Khövsgöl. Our species differs from the type material by a somewhat smaller width of valves (12–13 µm in the original description versus 11.2–11.8 µm in our material).

*Sellaphora khangalis* was found in the benthos of Lake Hangal and Lake Hoh [17]. We also found this species in Lake Khövsgöl and in an unnamed river. Our material is characterized by a smaller width of valves (9.5–10.0 μm for the type material versus 7.3–9.0 for our material). The species is known only from Mongolia.

*Sellaphora pseudopupula* was found in the Nuur *Sphagnum* bog [16,20]. It was shown by us in a nameless reservoir separated by a sandy spit from Lake Khövsgöl, a nameless lake and a nameless stream next to it, Lake Zavagitn Nur, and the Terkhiin River. Our material differs from the European material in a slightly smaller valve width (5.5–5.8 μm in our species versus 6–7 μm in the European population) [21]. This is a Holarctic species.

*Sellaphora perlaevissima* was previously shown in the benthos of the Barun Burh, Bayan, Jargalant, and Tsenher rivers [17]. It is illustrated by us in Lake Davaa and an unnamed reservoir separated by a sandy spit from Lake Khövsgöl, as well as from the Suman River. Our material is distinguished by a slightly higher stria frequency in 10 µm (15–16 for our material versus 13–15 for the type material). The species is known only from Mongolia.

*Sellaphora kusberi* was previously found in the benthos of the Tuul, Ynegtiin, Herlen rivers and Lake Hangal [17]. Our findings of this species are shown in lakes Davaa and Ögii, an unnamed reservoir separated by a sandy spit from Lake Khövsgöl, and two unnamed rivers. Our material as a whole is distinguished by a somewhat higher striae frequency in 10 µm (22–23 in our material versus 21–22 in the type material of this species) and also by a smaller width of valves (5.6–6.3 µm in our material versus 6.6 µm in the type material).

*Sellaphora intermissa* was previously shown in the benthos of the Ayagan Byrd, Bayan, Zuun Burh, and Jargalant Ynegtiin rivers [17]. We also found it in Lake Bayan and additionally in Khövsgöl Lake, Heeguer Bay, an unnamed lake near Khövsgöl Lake separated by a sandbar, Zavagitn Nur Lake, and in an unnamed river. The specimens illustrated by us generally have shorter valves (16.3–26.1 µm), while the type material is represented by longer valves (23–30 µm). In this regard, the width of the valves of our specimens is, on the whole, smaller than that of the type material (3.8–4.6 µm for our specimens versus 4.5–5.0 for the type material). The striae frequency in our material is somewhat higher than that for the type material (24–27 µm for our material versus 23–25 µm for the type material). The species is known only from Mongolia.

*Sellaphora seminulum* was previously shown in the sediments of Lake Davaa [17]. We illustrate this species from lakes Bayan and Khövsgöl. Our material, on the whole, fits into the accepted concept of this species. This is a Holarctic species.

### 3.2. First Findings for Mongolian Flora

*Sellaphora obesa* has been described from the UK [13]. This species was found by us in Mongolia and shown in an unnamed river flowing out of the Davaa Lake (periphyton) Khövsgöl Lake (benthos), an unnamed lake near Khövsgöl Lake separated by a sandbar (periphyton), and the Jargalant River (periphyton). Our material is morphologically close to the type material of this species. This species is known from the Holarctic [3].

*Sellaphora lanceolata* has been described from the UK [13]. This species was noted by us from the benthos of Khövsgöl Lake and sand from Terkhiin Tsagaan Lake. In general, our material is distinguished by a slightly smaller valve width, 6.6–7.8 µm, compared to the type material, 7.1–8.1 µm [13]. This species is a Holarctic species [3].

*Sellaphora pelagonica* was described from a dystrophic pond from North Macedonia [22]. We show this species from the benthos of different parts of Khövsgöl Lake, from the bottom sediment of an unnamed lake near Khövsgöl Lake separated by a sandbar, and from the periphyton of the Terkhiin River. The morphology of our findings is close to the two populations of species illustrated by Kochoska et al. [22].

*Sellaphora bisexualis* has been described from the UK [23]. We illustrate this view from Mongolia from the sediment from Lake Davaa, and also from an unnamed lake near Khövsgöl Lake separated by a sandbar. In general, our finding is distinguished by a slightly higher striae density (20–23 μm in our finding versus 18.5–21.5 in the type material of *S. bisexualis*).

*Sellaphora medioconvexa* is known from Europe [24,25,26]. We show this species from the benthos and bottom sediments of Davaa Lake, as well as from the periphyton of an unnamed river flowing out of the Davaa Lake.

*Sellaphora digna* has been described from Germany [15,27,28]. We illustrate this species from the sediment and sand of Davaa Lake and from an unnamed lake near Khövsgöl Lake separated by a sandbar.

*Sellaphora disjuncta* has been described from Germany [29]. The species is also known from North America [30] and from the European part of Russia [31]. From Mongolia, we illustrate this species from the periphyton of an unnamed river flowing out of the Davaa Lake, from the sediments of an unnamed lake near Khövsgöl Lake separated by a sandbar, from the phytoplankton of an unnamed lake near the valley of the river flowing into Khövsgöl Lake, as well as from the benthos of the unnamed river. On the basis of striae density values, our findings are closest to North American material of this species (24–25 in 10 µm in our findings versus 23–28 in 10 µm in the North American material of the species), while the material from Lake Glubokoe (Russia) has a lower striae density (21–23 in 10 µm).

*Sellaphora atomoides* was described as *Navicula atomoides* from Antwerp, Belgium [32]. In Mongolia, we show this species in the unnamed lake separated from Khövsgöl Lake by a sandbar (sediments) and the benthos of Lake Terkhiin Tsagaan. It is a rather heterogeneous taxon that is poor in morphological features [31]. It belongs to a widespread species [3].

*Sellaphora pseudoventralis* has been described as *Navicula pseudoventralis* from Germany, Großer Madebrökensee [33,34]. We found this species in the bottom sediment and the benthos of Lake Davaa. The species is a widespread, probably cosmopolitan species [35]. In general, our finding corresponds to another population of this species from Lake Glubokoe and European material [21,31,35].

*Sellaphora cosmopolitana* was originally described as *Navicula arvensis* var. *major* Lange-Bertalot in Krammer and Lange-Bertalot [36]. We found this species in the benthos of a puddle with mosses next to the Davaa Lake. The species is known from Ecuador [37] and Europe [36].

*Sellaphora stroemii* was described as *Navicula stroemii* from Norway [38]. In Mongolia, we show this species from the Jargalant River periphyton. In general, our finding morphologically corresponds to the European population of this species shown by Cantonati et al. [21].

*Sellaphora multiconfusa* has been described as *Naviculadicta multiconfusa* from James Lake, Canada [39]. We have shown this species in the benthos and bottom sediments of Davaa Lake, as well as from the bottom sediments of an unnamed lake near Khövsgöl Lake separated by a sandbar. The species is a Holarctic species known from Canada and Europe [39]. Our findings are morphologically close to the type material of this species [39].

*Sellaphora vekhovii* has been described as *Naviculadicta vekhovii* from the Yugorskiy Shar Peninsula, Siberia [40]. We illustrate this species from the bottom sediments of Lake Davaa, from the periphyton of an unnamed river flowing out of the Davaa Lake, from the benthos of a puddle with moss next to the Davaa Lake, from the periphyton of an unnamed lake near the Terkhiin Tsagaan Lake, and also from a number of nameless rivers. The species is distributed in the European part of Russia, the far north of Western Siberia, and the Baikal region [41]. Our findings are morphologically close to the type material of this species [40].

*Sellaphora glomus* comb. nov. has been described from Scotland as *Navicula glomus* [42]. It was found by us in the bottom sediments of an unnamed lake near Khövsgöl Lake separated by a sandbar. The species is a Holarctic species known from the Shetland Islands, Alps, Yamal Peninsula, and Russia [25,43]. Our findings are morphologically consistent with the diagnosis given by Krammer and Lange-Bertalot [25].

*Sellaphora dorofeyukae* sp. nov., according to electron microscopic examination, belongs to a morphologically interesting group of species known from Asia and the tropics of the New World. These species are characterized by significant external massive silicification of valves, which is unknown for described groups of *Sellaphora* sensu Mann et al. [14]. These species are known from both ancient Lake Baikal (*S. amicula* Kulikovskiy, Metzeltin and Lange-Bertalot, *S. psedamicula* Kulikovskiy, Metzeltin and Lange-Bertalot, *S. ovalis* Kulikovskiy, Metzeltin and Lange-Bertalot) and tropical streams in South America (*S. lambda* (Cleve) Metzeltin and Lange-Bertalot, *S. renata* Metzeltin and Lange-Bertalot, etc.). A brief description of the new species and morphologically related species of *Sellaphora* is given by us in Table 2.

A separate mention should be made for the genus *Okhapkinia* Glushchenko, Kulikovskiy and Kociolek in Kulikovskiy et al., described by us from Southeast Asia and attributed to representatives of the family Sellaphoraceae. A representative of the genus has, in addition to the characteristic generic characters, massive silicification of the valves from the outer surface [4].

The thickness of this silica layer is different; for example, in *Okhapkinia* and tropical *Sellaphora*, it is developed to a greater extent than in Baikal *Sellaphora*. A thick silicified layer from the outer surface of the previously mentioned *Sellaphora* species has wide openings (hereinafter referred to as “elliptical lacunae” after Metzeltin and Lange-Bertalot) [46] and has a shape from rounded to elongated, at the bottom of which there are separate areolae, from one to several [4], which, according to Metzeltin and Lange-Bertalot [46], together form a system of holes of the first and second order. In the case of *Okhapkinia*, the areolae are crater-shaped and separated by ridge-like thickenings, which was not noted for *Sellaphora* [4,47]. In general, the presence of species and even genera (for example, *Altana*, *Placoneis*) with heavily silicified valves is typical of tropical regions and ancient lakes [8,47].

*Sellaphora dorofeyukae* sp. nov. has the greatest morphological similarity with the Baikal species *S. amicula* and *S. pseudoamicula* (see Table 2). *Sellaphora dorofeyukae* sp. nov. have a common raphe-sternum structure (the presence of a conopeum and two parallel apical depressions), a similar width of the central and axial areas, and a similar width of valves (16.6–18.4 µm in *S. dorofeyukae* sp. nov. versus 17.3–21.3 µm in *S. amicula*) as well as a similar shape and localization of lacunae on the outer surface of the valves (see Table 2). The number of areolae in each of the lacunae is also similar (from 2 to 10 areolae in *S. dorofeyukae* sp. nov. versus 2–11 areoles in *S. amicula*). The species differ in valve shape: In *S. dorofeyukae* sp. nov., the shape is linear with parallel margins and widely rounded ends. In *S. amicula*, the shape ranges from linear to linear-elliptical with slightly convex margins and widely rounded valve ends (Table 2). Striae density in *S. dorofeyukae* sp. nov. is 16–19 in 10 µm, and in *S. amicula* striae density is 20–22 in 10 µm (Table 2). The density of areolae also varies between species. In Kulikovskiy et al. [8], when describing the species the density of areolae was taken to indicate first-order areolae (according to Metzeltin and Lange-Bertalot) [46], not separately taking into account the areolae located in lacunae (second-order areolae, according to Metzeltin and Lange-Bertalot) [46]. This indicator is also similar to ours (15–25 areolae in 10 microns). Our observations show that the density of areolae lying in lacunae (second-order areolae (according to Metzeltin and Lange-Bertalot) [46] in the central region of the valve in *S. amicula* is approximately 38 in 10 µm [8] (Pl. 98, Figures 1 and 2). In *S. dorofeyukae* sp. nov., the density of second-order areolae is more variable in different parts of the valve: 50 areolae in 10 µm in the central part of the valve, up to 70 areolae in 10 µm near the central area, and at the ends of the valve, there are 40–45 areolae in 10 µm (Table 2).

*S. pseudamicula* as *S. amicula* are also morphologically close to *S. dorofeyukae* sp. nov. (Table 2). At the same time, *S. dorofeyukae* sp. nov. differs by its narrower raphe-sternum (3 µm in *S. dorofeyukae* sp. nov. versus 4.0–4.5 µm in *S. pseudamicula*) as well as a narrower valve width (16.6–18.4 µm in *S. dorofeyukae* sp. nov. versus 22.7–23.3 µm in *S. pseudamicula*). The density of areolae, according to Kulikovskiy et al. [8] (first-order areolae, according to Metzeltin and Lange-Bertalot) [46], is 15–20 in 10 µm (Table 2). The same data for *S. dorofeyukae* sp. nov. are 15–25 in 10 µm.

*Sellaphora krsticii* is described from St. Naum springs, Lake Ohrid [44], close to *S. dorofeyukae* sp. nov. Both species have a conopeum, distinct lacunae, and similar valve widths (13.5–20.0 µm in *Sellaphora krsticii* versus 16.6–18.4 µm in *S. dorofeyukae* sp. nov.). At the same time, the species differ in the shape of the valves (linear to linear-lanceolate with obtusely rounded, unattenuated ends, versus linear with parallel edges and widely rounded ends in *S. dorofeyukae* sp. nov.). The ultrastructure of the species is also radically different: *Sellaphora krsticii* lacks a siliceous layer pierced by holes, at the bottom of which areolae lie (areolae of the first and second orders, according to Metzeltin and Lange-Bertalot). The areolae density in *S. krsticii* is 40–42 in 10 µm, while in *S. dorofeyukae* sp. nov. the second-order areolae density is 15–25 in 10 µm (Table 2). In *S. krsticii*, the only elongated areolae are located near the central area, but they do not have a lacuna structure (Table 2).

*Sellaphora lambda* is similar in its ultrastructure to *S. dorofeyukae* sp. nov. Both species have similar features such as the structure of the raphe-sternum and the presence of first- and second-order areolae. The density of areolae is close between the species, 16–19 in *S. dorofeyukae* sp. nov. versus 18–20 at the ends and 13–16 striae in the central part in *S. lambda*. The number of first-order areolae (visible in the LM) in *S. lambda* is 14–15 in 10 µm; in *S. dorofeyukae* sp. nov. these data are more variable, 15–25 in 10 µm. This range is due to the alternation of lacunae and individual areolae, which is clearly seen in SEM (Figure 7). The number of second-order areolae (located in lacunae) is also similar in the species: 2–9 areolae in *S. lambda* versus 2–10 areolae in *S. dorofeyukae* sp. nov. At the same time, the outer silica layer in *S. lambda* is more pronounced than in *S. dorofeyukae* sp. nov. The shapes of the valves of the species also differ; *S. lambda* has linear valves with slightly concave margins and widely rounded ends, while *S. dorofeyukae* sp. nov. has linear valves with parallel edges and widely rounded ends (Table 2). The density of the areolae of the basal layer (second-order areolae) is different between species; in *S. lambda*, these data can be given only for an unknown part of the valve [46] (p. 402, Taf. 84, Figure 3) and are 40–50 in 10 µm (Table 2). In *S. dorofeyukae* sp. nov., the density of second-order areolae is more variable in different parts of the valve: 50 areolae in 10 µm in the central part of the valve, up to 70 areolae in 10 µm near the central area, and 40–45 areolae in 10 µm at the ends of the valve (Table 2).

Thus, the number of *Sellaphora* known from the aquatic ecosystems of Mongolia is 45 species. Of course, even this number of species will not reflect the complete composition of the genus in Mongolia. Additional data on the distribution of species already known in the Mongolian flora, including those known only from the type locality, have been obtained.

Further research is needed to add new localities of both previously known species for the country and to identify new species. It is necessary to conduct molecular genetic studies that will allow a greater degree of comparison between European and Asian populations of *Sellaphora* species.

## 4. Materials and Methods

### 4.1. Sampling

Samples from Mongolia were collected by M.S. Kulikovskiy in 2015. Physical and chemical water parameters were measured with a Hanna Combo (HI 98129) multiparameter probe (Hanna Instruments Ltd., Inc., New York, NY, USA). Samples had been collected from different parts of the bay and from different substrates. A list of slides and their characteristics is given in Table 3.

### 4.2. Preparation of Slides and Microscope Investigation

Samples have been processed by means of a standard procedure involving treatment with 10% HCl and concentrated hydrogen peroxide. After treatment with HCl, the sample was washed with deionized water. Permanent diatom preparations were mounted in Naphrax^®^ (Brunel Microscopes Ltd., Chippenham, UK; refractive index = 1.73) [47]. Light microscopic (LM) observations were performed by means of a Zeiss Axio Scope A1 (Carl Zeiss Microscopy GmbH, Göttingen, Germany) microscope equipped with an oil immersion objective (×100/n.a.1.4, Nomarski differential interference contrast, DIC) and a Zeiss Axio Cam ERc 5s camera (Carl Zeiss NTS Ltd., Oberkochen, Germany). The valve ultrastructure was examined by means of a JSM-6510LV 6510LV scanning electron microscope (JEOL Ltd., Tokyo, Japan) operated at 10 kV and 11 mm working distance. For scanning electron microscopy (SEM), parts of the suspensions were fixed on aluminum stubs after air drying. The stubs were sputter-coated with 50 nm of gold in an Eiko IB 3 (Eiko Engineering Co., Ltd., Hitachinaka, Japan).

Samples and slides are deposited in the collection of Maxim Kulikovskiy at the herbarium of the Institute of Plant Physiology Russian Academy of Sciences, Moscow, Russia.

## Figures and Tables

**Table 1:** Species of the genus *Sellaphora* previously described and/or recorded in Mongolia according to Dorofeyuk and Kulikovskiy [17].

Species	Type Locality	Distribution in Mongolia
*Sellaphora auldreekie* D.G. Mann and S.M. McDonald in D.G. Mann et al. 2004 [14]	United Kingdom	Surface sediments of the Great Lakes Depression, *sine loco*
*Sellaphora bacilliformis* (Grunow) Mereschkowsky 1902	Norway, Dovre	Khövsgöl Lake, benthos; Cheltyge and Hortak Lakes, plankton
*Sellaphora bacillum* (Ehrenberg) D.G. Mann 1989	Africa, Algeria	Achit, Dund, Hoton, Dood, Hodoo, Davaa, Terhiyn Tsagaan, Gun, Hangal, Buyr Lakes, bottom sediments; Har, Har Us Bayan Lakes, bottom sediments; Khövsgöl Lake, benthos and bottom sediments; Changa River, plankton; Olon and Hag lakes, plankton; Tuul River, benthos; Small ponds of Buyr Lake catchment area, benthos and epiphyton; Surface sediments of the Great Lakes Depression, *sine loco*
*Sellaphora boltziana* Metzeltin, Lange-Bertalot and Nergui 2009	Mongolia	Hoh lake, benthos
*Sellaphora bullata* (Østrup) Lange-Bertalot and Metzeltin in Metzeltin et al. 2009	Denmark	Haraa and Tuul Rivers, benthos; Ayagan Byrd, Ynegtiin, Herlen, Tsenher, Shurguit, Jargalantl Rivers and Hoh Lake, benthos
*Sellaphora ellipticolanceolata* Metzeltin, Lange-Bertalot and Nergui 2009	Mongolia	Tsenkher River
*Sellaphora fennica* Lange-Bertalot and Metzeltin in Metzeltin et al. 2009	Mongolia	Haraa and Tuul Rivers, benthos; Ayagan Byrd, Ynegtiin, Herlen, Tsenher, Shurguit, Jargalant Rivers and Hoh Lake, benthos
*Sellaphora hustedtii* (Krasske) Lange-Bertalot and Werum 2004	Germany, Hesse	Terhiyn Tsagaan and Davaa Lakes, bottom sediments; Tuul River, plankton
*Sellaphora intermissa* Metzeltin, Lange-Bertalot and Nergui 2009	Mongolia	Ayagan Byrd, Ynegtiin, Herlen, Tsenher, Shurguit, Jargalant Rivers and Hoh Lake, benthos
*Sellaphora interrupta* Metzeltin, Lange-Bertalot and Nergui 2009	Mongolia	Bayan and Jargalant Rivers, benthos
*Sellaphora khangalis* Metzeltin and Lange-Bertalot in Metzeltin et al. 2009	Mongolia	Hangal Lake, benthos
*Sellaphora kretschmeri* Metzeltin, Lange-Bertalot and Nergui 2009	Mongolia	Hoh Lake, benthos
*Sellaphora kusberi* Metzeltin, Lange-Bertalot and Nergui 2009	Mongolia	Tuul, Ynegtiin, Herlen Rivers and Hangal Lake, benthos
*Sellaphora mongolocollegarum* Metzeltin and Lange-Bertalot in Metzeltin et al. 2009	Mongolia	Tuul, Ynegtiin, Shurguit, Jargalant Rivers and Hangal Lake, benthos
*Sellaphora mutata* (Krasske) Lange-Bertalot in Lange-Bertalot et Metzeltin 1996	Germany, Saxony	Hag Lake, plankton; Urd Tamir River, plankton; Small ponds of Buyr Lake catchment area, benthos and epiphyton
*Sellaphora parapupula* Lange-Bertalot in Lange-Bertalot et Metzeltin 1996	Europe	Achit, Khövsgöl, Terhiyn Tsagaan, Buyr Lakes, bottom sediments; Hogon lake, plankton
*Sellaphora perlaevissima* Metzeltin, Lange-Bertalot and Nergui 2009	Mongolia	Barun Burh, Bayan, Jargalant, Tsenher Rivers, benthos
*Sellaphora permutata* Metzeltin, Lange-Bertalot and Nergui 2009	Mongolia	Herlen River, benthos
*Sellaphora perobesa* Metzeltin, Lange-Bertalot and Nergui 2009	Mongolia	Har Us River, benthos
*Sellaphora pseudobacillum* (Grunow) Lange-Bertalot and Metzeltin in Metzeltin et al. 2009	Mongolia	Khövsgöl Lake and Changa River, plankton and benthos; Hangal Lake and Tuul River, benthos
*Sellaphora pseudopupula* (Krasske) Lange-Bertalot in Lange-Bertalot et al. 1996	Germany, Hesse	*Sphagnum* bog Nur
*Sellaphora pupula* (Kützing) Mereschkovsky 1902	Germany, near Nordhausen	Achit, Dund, Hoton Har, Har Us, Bayan, Khövsgöl, Terhiyn Tsagaan, Dava, Hodoo, Gun, Hangal, Buyr Lakes and Bog Nur, bottom sediments; Hoton, Nogon, Olon, Ust, Hag, Ogiy, Sangiyn Dalay Lakes, plankton; Ongi, Chatschim, Hortak, Tamir, Tuul Rivers, plankton Surface sediments of the Great Lakes Depression, *sine loco*; Loamy takyr desert soils; Dood Lake, plankton and bottom sediments
*Sellaphora rectangularis* (W. Gregory) Lange-Bertalot and Metzeltin 1996	Scotland, Isle of Mull	Achit, Dund, Hoton, Har, Har Us, Dood, Khövsgöl, Terhiyn Tsagaan, Hodo, Davaa, Gun, Hangal, Buyr Lakes, bottom sediments; Hag Lake, plankton
*Sellaphora rostrata* (Hustedt) J.R. Johansen 2004	Germany, Bremen	Khövsgöl, Davaa, Buyr Lakes, bottom sediments
*Sellaphora schrothiana* Metzeltin, Lange-Bertalot and Nergui 2009	Mongolia	Braun Burh, Herlen, Tsenher Rivers and Hangal, Hoh Lakes, bottom sediments
*Sellaphora seminulum* (Grunow) D.G. Mann 1989	Austria	Davaa Lake, bottom sediments
*Sellaphora simillima* Metzeltin, Lange-Bertalot and Nergui 2009	Mongolia	Hoh Lake, benthos
*Sellaphora stauroneioides* (Lange-Bertalot) Veselá and J.R. Johansen 2009	Finland	*Sphagnum* Bog Nur
*Sellaphora vitabunda* (Hustedt) D.G. Mann 1989	Germany, Holstein	Achit, Dood, Khövsgöl, Terhiyn Tsagaan, Davaa, Gun, Hangal, Buyr, bottom sediments; Surface sediments of the Great Lakes Depression, *sine loco*
*Sellaphora wittrockii* (Lagerstedt) Lange-Bertalot and Metzeltin 2009	Norway, Svalbard	Bayan River, benthos

**Table 2:** Comparison of morphological features of *Sellaphora dorofeyukae* sp. nov. and related species.

	*S. dorofeyukae* sp. nov.	*S. amicula*	*S. pseudamicula*	*S. krsticii*	*S. lambda*
Valve shape	Linear with parallel margins and broadly rounded ends	Linear but linear-elliptical, all with convex margins and broadly rounded ends	Linear to linear-elliptical with convex margins and broadly rounded ends	Linear to linear lanceolate with bluntly rounded, not protracted ends	Linear with weakly concave margins and broadly rounded ends
Axial area and conopeum	Encompassing the complex of raphe-sternum, flaps of the conopeum, and parallel apical depressions and is straight (not undulating in parts), ca. 3 μm broad	Encompassing the complex of raphe-sternum, flaps of the conopeum, and parallel apical depressions and is straight (not undulating in parts), ca. 3 μm broad	Including conopeum and parallel apical depressions, 4.0–4.5 μm broad	Narrow and linear. Distinct conopeum present	Narrow and linear. Distinct conopeum present *
Central area	Apically broad elliptical, extended to 1/3–1/4 of the valve breadth	Apically broad elliptical, extended to 1/4–1/3 of the valve breadth	Apically broad elliptical, extended to 1/3 of the valve breadth	Elliptical, 1/3 to 1/2 of valve breadth.	Apically broad elliptical, extended to 1/2–1/3 of the valve breadth *
Valve length (μm)	52.1–77.8	44.7–103.0	91.3–96.7	52–100	45–140
Valve breadth (μm)	16.6–18.4	17.3–21.3	22.7–23.3	13.5–20.0	15–21
Striae number in 10 μm	16–19	20–22	16–17	14–16	13–16 at the central part, 18–20 near the ends
The shape of the lacunae and their location on the valve	Elongated lacunae on both sides of the conopeum; On the valve face, the lacunae are shorter, alternating with small areolae; Closer to the valve mantle, the lacunae are elongated; At the valve ends, the lacunae are almost not expressed	Elongated lacunae on both sides of the conopeum; On the valve face, the lacunae are shorter, alternating with small areolae; Closer to the valve mantle, the lacunae are more elongated; At the valve ends, the lacunae are almost not expressed	n.d.	There are no visible lacunae; in the conopeum, the poroids are somewhat elongated (mainly in the central part of the valve)	Elongated lacunae on both sides of the conopeum, especially near the central area; On the valve face, the lacunae are shorter, alternating with small areolae; At the valve ends, the lacunae are almost not expressed
Perforations of the outer layer of silica (= “First order areolae”, “lacunae” by Metzeltin and Lange-Bertalot; = “areolae” from other authors), in 10 μm	15–25	15–25	15–20	40–42 **	14–15 *
Areolae basal layer (=“Second order areolae” by Metzeltin and Lange-Bertalot) number in 10 μm	In lacunae in the central part of the valve: 50; up to 70 in lacunae located near the central area and 40–45 at the valve ends.	≈38 at the central part of the valve	n.d.	n.d.	40–50 *
Number of areolae basal layer (in each of the lacunae, = “Second order areolae” by Metzeltin and Lange-Bertalot)	2–10	2–11 *	n.d.	n.d.	2–9 *
Distribution	Mongolia, Davaa Lake	Russia, Baikal Lake	Russia, Baikal Lake	North Macedonia, St. Naum springs, Lake Ohrid	South America, Guyana, Demerara River and Essequibo River
References	This study	[8]	[8]	[44,45]	[46]

* counted from published data; ** given the meaning of ordinary areolae; n.d.—no data.

**Table 3:** List of the collected samples and their characteristics.

Sample No.	Slide No.	Locality	Coordinates	Substratum	Cond., µS/cm	pH	t, °C	Collection of Date
Mn079	02431	Davaa Lake	48°10.803′ N; 98°46.107′ E	benthos	20	8.1	13	8 July 2015
Mn085	02437	Davaa Lake	48°10.803′ N; 98°46.107′ E	periphyton	20	8.1	13	8 July 2015
Mn088	02440	Davaa Lake	48°10.827′ N; 98°45.828′ E	benthos, sand	20	6.9	13	8 July 2015
Mn089	02441	Davaa Lake	48°10.827′ N; 98°45.828′ E	benthos, sand	20	6.9	13	8 July 2015
Mn092	02444	Davaa Lake	48°10.827′ N; 98°45.828′ E	periphyton	20	6.9	13	8 July 2015
Mn095.1	02447	Davaa Lake	48°11.145′ N; 98°45.746′ E	bottom sediment a depth of 4 m, column depth 15 cm	38	7.7	15	8 July 2015
Mn095.5	02452	Davaa Lake	48°11.145′ N; 98°45.746′ E	bottom sediment a depth of 4 m, column depth 4 cm	38	7.7	15	8 July 2015
Mn104	02457	Unnamed river flowing out of the Davaa Lake	48°10.791′ N; 98°46.272E′	periphyton	38	7.7	15	8 July 2015
Mn101	02458	Puddle with moss next to the Davaa Lake	48°10.815′ N; 98°45.835′ E	benthos	124	6.73	14	8 July 2015
Mn156	02463	Bayan Lake, small bay	48°26.668′ N; 95°10.937′ E	benthos	260	9.8	10.5	12 July 2015
Mn161	02474	Bayan Lake, small bay	48°26.668′ N; 95°10.937′ E	benthos	260	9.8	10.5	12 July 2015
Mn163	02475	Bayan Lake, small bay	48°26.668′ N; 95°10.937′ E	benthos	260	9.8	10.5	12 July 2015
Mn164	02476	Bayan Lake, small bay	48°26.668′ N; 95°10.937′ E	benthos, sand	260	9.8	10.5	12 July 2015
Mn166	02478	Bayan Lake, small bay	48°26.668′ N; 95°10.937′ E	benthos, sand	260	9.8	10.5	12 July 2015
Mn282	02605	Khövsgöl Lake	50°59.380′ N; 100°42.507′ E	benthos	236	8.7	11.5	21 July 2015
Mn285	02611	Unnamed lake near Khövsgöl Lake, separated by a sandbar	50°59.165′ N; 100°42.514′ E	periphyton	100	8.9	18	28 July 2015
Mn290	02620	Unnamed river	50°56.140′ N; 100°45.585′ E	benthos	42	7.3	9	28 July 2015
Mn232	02621	Khövsgöl Lake	50°59.380′ N; 100°42.507′ E	periphyton	157	9.6	20	18 July 2015
Mn287	02622	Unnamed lake near Khövsgöl Lake, separated by a sandbar	50°59.165′ N; 100°42.514′ E	periphyton	100	8.9	18	28 July 2015
Mn289.3	02636	Unnamed lake near Khövsgöl Lake, separated by a sandbar	50°59.165′ N; 100°42.514′ E	surface sediment a depth of 5 m	100	8.9	18	28 July 2015
Mn289.1	02642	Unnamed lake near Khövsgöl Lake, separated by a sandbar	50°59.165′ N; 100°42.514′ E	bottom sediment a depth of 5 m, column depth 20 cm	100	8.9	18	28 July 2015
Mn329	02675	Unnamed lake near the valley of the river flowing into Khövsgöl Lake	50°45.290′ N; 100°30.871′ E	phytoplankton	228	9.6	20.5	22 July 2015
Mn335.8	02684	Khövsgöl Lake, Heeguer Bay	50°38.641′ N; 100°31.397′ E	benthos	304	10.6	22	22 July 2015
Mn307	02687	Khövsgöl Lake, Heeguer Bay	50°38.641′ N; 100°31.397′ E	periphyton	304	10.6	22	22 July 2015
Mn313	02696	Khövsgöl Lake, Heeguer Bay	50°38.641′ N; 100°31.397′ E	periphyton	304	10.6	22	22 July 2015
Mn236a	02984	Unnamed lake near Khövsgöl Lake, separated by a sandbar	50°48.083′ N; 100°14.631′ E	periphyton	155	9.5	20	18 July 2015
Mn272	03005	Unnamed lake near Khövsgöl lake, separated by a sandbar	50°39.220′ N; 100°15.165′ E	phytoplankton	230	9.7	23	18 July 2015
Mn283.2	03009	Khövsgöl Lake, Boreug Bay	50°59.380′ N; 100°42.507′ E	benthos	236	8.7	11.5	21 July 2015
Mn292	03015	Unnamed river	50°50.519′ N; 100°45.585′ E	benthos, sand	90	7.4	6.5	28 July 2015
Mn293	03016	Unnamed river	50°50.519′ N; 100°45.585′ E	periphyton	90	7.4	6.5	28 July 2015
Mn294	03017	Unnamed river	50°50.519′ N; 100°45.585′ E	periphyton	90	7.4	6.5	28 July 2015
Mn040	03027	Unnamed lake near Terkhiin Tsagaan Lake	48°08.496′ N; 99°49.823′ E	periphyton	94	8	14	6 July 2015
Mn051	03030	Terkhiin Tsagaan Lake	48°08.322′ N; 99°49.745′ E	benthos	140	8.8	16	6 July 2015
Mn147	03053	Holbo Lake	48°34.507′ N; 95°25.679′ E	periphyton	346	10.1	18	11 July 2015
Mn034	03097	Suman River	48°06.166′ N; 99°57.621′ E	benthos, sand	100	9	4.5	6 July 2015
Mn058	03101	Terkhiin Tsagaan Lake	48°09.551′ N; 99°42.984′ E	benthos, sand	143	8.4	14	6 July 2015
Mn063	03105	Terkhiin Tsagaan Lake	48°07.574′ N; 99°36.852′ E	benthos	142	9	16	6 July 2015
Mn192	03121	Unnamed lake	49°05.716′ N; 96°40.276′ E	benthos	384	7.7	10	15 July 2015
Mn195	03123	Unnamed spring near the lake	49°05.816′ N; 96°40.032′ E	benthos	111	7.1	8	15 July 2015
Mn006	03149	Ögii Lake	47°47.240′ N; 102°45.431′ E	benthos	541	9.2	23.4	5 July 2015
Mn008	03150	Ögii Lake	47°47.240′ N; 102°45.431′ E	benthos, sand	541	9.2	23.4	5 July 2015
Mn009	03151	Ögii Lake	47°47.240′ N; 102°45.431′ E	periphyton	541	9.2	23.4	5 July 2015
Mn117	03163	Zavagan Lake	48°08.196′ N; 98°49.710′ E	benthos	250	8.4	15.6	9 July 2015
Mn119.3	03167	Zavagan Lake	48°08.196′ N; 98°49.710′ E	bottom sediment a depth of 5 m, column depth 1 cm	250	8.4	15.6	9 July 2015
Mn122	03169	Terkhiin River	48°04.940′ N; 98°55.078′ E	periphyton	146	8.6	16	9 July 2015
Mn224	03183	Jargalant River	49°37.376′ N; 99°35.059′ E	periphyton	198	8.6	21	17 July 2015

## Data Availability

Not applicable.

## References

[1] Mereschkowsky C. (1902). On *Sellaphora*, a new genus of diatoms. Ann. Mag. Nat. Hist..

[2] Kociolek J.P., Blanco S., Coste M., Ector L., Liu Y., Karthick B., Kulikovskiy M., Lundholm N., Ludwig T., Potapova M. DiatomBase.

[3] Kulikovskiy M.S., Glushchenko A.M., Genkal S.I., Kuznetsova I.V. (2016). Identification Book of Diatoms from Russia.

[4] Kulikovskiy M.S., Glushchenko A.M., Kuznetsova I.V., Kociolek J.P. (2018). Description of the new freshwater diatom genus *Okhapkinia* gen. nov. from Laos (Southeast Asia), with notes on family Sellaphoraceae Mereschkowsky 1902. Fottea.

[5] Guiry M.D., Guiry G.M. *AlgaeBase*. *World-Wide Electronic Publication*, National University of Ireland, Galway. http://www.algaebase.org.

[6] Schimani K., Abarca N., Zimmermann J., Skibbe O., Jahn R., Kusber W.-H., Leya T., Mora D. (2023). Molecular phylogenetics coupled with morphological analyses of Arctic and Antarctic strains place *Chamaepinnularia* (Bacillariophyta) within the Sellaphoraceae. Fottea.

[7] Li Y., Metzeltin D., Gong Z. (2010). Two new species of *Sellaphora* (Bacillariophyta) from a deep oligotrophic plateau lake, Lake Fuxian in subtropical China. Chin. J. Oceanol. Limnol..

[8] Kulikovskiy M.S., Lange-Bertalot H., Metzeltin D., Witkowski A. (2012). Lake Baikal: Hotspot of endemic diatoms I. Iconogr. Diatomol..

[9] You Q.-M., Kociolek J.P., Cai M.-J., Lowe R.L., Liu Y., Wang Q.-X. (2017). Morphology and ultrastructure of *Sellaphora constrictum* sp. nov. (Bacillariophyta), a new diatom from southern China. Phytotaxa.

[10] Liu Y., Kociolek J.P., Lu X., Fan Y. (2020). A new *Sellaphora* Mereschkowsky species (Bacillariophyceae) from Hainan Island, China, with comments on the current state of the taxonomy and morphology of the genus. Diatom Res..

[11] Glushchenko A., Kezlya E., Maltsev Y., Genkal S., Kociolek J.P., Kulikovskiy M. (2022). Description of the Soil Diatom *Sellaphora terrestris* sp. nov. (Bacillariophyceae, Sellaphoraceae) from Vietnam, with Remarks on the Phylogeny and Taxonomy of *Sellaphora* and Systematic Position of *Microcostatus*. Plants.

[12] Ni H.-P., Guo J.-S., Tang Z.-B., Huang Y.-Y., Kociolek J.P., Li Y.-L. (2022). *Sellaphora tanghongquii* sp. nov. (Bacillariophyta, Sellaphoraceae), a new diatom species from the Southern China. Phytotaxa.

[13] Mann D.G., McDonald S.M., Bayer M.M., Droop S.J.M., Chepurnov V.A., Loke R.E., Ciobanu A., du Buf J.M.H. (2004). The *Sellaphora pupula* species complex (Bacillariophyceae): Morphometric analysis, ultrastructure and mating data provide evidence for five new species. Phycologia.

[14] Mann D.G., Thomas S.J., Evans K.M. (2008). Revision of the diatom genus *Sellaphora*: A first account of the larger species in the British Isles. Fottea.

[15] Wetzel C.E., Ector L., Van de Vijver B., Compère P., Mann D.G. (2015). Morphology, typification and critical analysis of some ecologically important small naviculoid species (Bacillariophyta). Fottea.

[16] Kulikovskiy M.S., Dorofeyuk N.I. (2010). New diatoms for the Mongolian flora. Nov. Sist. Nizshikh Rastenii.

[17] Dorofeyuk N.I., Kulikovskiy M.S. (2012). Diatoms of Mongolia. Biological Resources and Natural Conditions of Mongolia: Proceedings of Joint Russian-Mongolian Complex Biological Expeditions RAS and MAS.

[18] Metzeltin D., Lange-Bertalot H., Nergui S. (2009). Diatoms in Mongolia. Iconogr. Diatomol..

[19] Metzeltin D., Witkowski A. (1996). Diatomeen der Bären-Insel Süsswasser und marine Arten. Iconogr. Diatomol..

[20] Kulikovskiy M.S., Lange-Bertalot H., Witkowski A., Dorofeyuk N.I., Genkal S.I. (2010). Diatom assemblages from *Sphagnum* bogs of the world. I. Nur bog in northern Mongolia. Bibl. Diatomol..

[21] Cantonati M., Kelly M.G., Lange-Bertalot H. (2017). Freshwater Benthic Diatoms of Central Europe: Over 800 Common Species Used in Ecological Assessment.

[22] Kochoska H., Zaova D., Videska A., Mitic-Kopanja D., Naumovska H., Wetzel C.E., Ector L., Levkov Z. (2021). *Sellaphora pelagonica* (Bacillariophyceae), a new species from dystrophic ponds in the Republic of North Macedonia. Phytotaxa.

[23] Mann D.G., Evans K.M., Chepurnov V.A., Nagai S. (2009). Morphology and formal description of *Sellaphora bisexualis* sp. nov. (Bacillariophyta). Fottea.

[24] Hustedt F., Rabenhorst L. (1961). Die Kieselalgen Deutschlands, Österreichs und der Schweiz unter Berücksichtigung der übrigen Länder Europas sowie der Angrenzenden Meeresgebiete. Bd. VII: Teil 3: Lieferung 1. Rabenhorst’s Kryptogamen Flora von Deutschland, Österreich und der Schweiz.

[25] Krammer K., Lange-Bertalot H., Ettl H., Gerloff J., Heynig H., Mollenhauer D. (1986). Bacillariophyceae. Teil 1. Naviculaceae. Süßwasserflora von Mitteleuropa, Bd. 2/1.

[26] Genkal S.I., Shelekhova T.S., Komulaynen S.F. (2021). Diatoms in the algal cenoses of Lamba lake (Petrozavodsk, Repubilic of Karelia). Bot. Zhurnal.

[27] Hustedt F. (1957). Die Diatomeenflora des Fluß-systems der Weser im Gebiet der Hansestadt Bremen. Abh. Naturwissenschaftlichen Ver. Brem..

[28] Heudre D., Wetzel C.E., Moreau L., Ector L. (2018). *Sellaphora davoutiana* sp. nov.: A new freshwater diatom species (Sellaphoraceae, Bacillariophyta) in lakes of Northeastern France. Phytotaxa.

[29] Schmidt A. (1930). Atlas der Diatomaceen-Kunde.

[30] Polaskey M., Ripple H. *Sellaphora disjuncta*. In Diatoms of North America. https://diatoms.org/species/sellaphora_disjuncta.

[31] Chudaev D.A., Gololobova M.A. (2016). Diatom Algae in Glubokoe Lake (Moscow Region).

[32] Van Heurck H. (1880). Synopsis des Diatomées de Belgique. Atlas.

[33] Schmidt A. (1936). Atlas der Diatomaceen-Kunde.

[34] Hustedt F. (1953). Diatomeen aus dem Naturschutzgebiet Seeon. Arch. Hydrobiol..

[35] Chudaev D., Gololobova M. (2015). *Sellaphora smirnovii* (Bacillariophyta, Sellaphoraceae), a new small-celled species from Lake Glubokoe, European Russia, together with transfer of *Navicula pseudoventralis* to the genus *Sellaphora*. Phytotaxa.

[36] Krammer K., Lange-Bertalot H. (1985). Naviculaceae Neue und wenig bekannte Taxa, neue Kombinationen und Synonyme sowie Bemerkungen zu einigen Gattungen. Bibl. Diatomol..

[37] Rumrich U., Lange-Bertalot H., Rumrich M. (2000). Diatoms of the Andes. From Venezuela to Patagonia/Tierra del Fuego and two additional contributions. Iconogr. Diatomol..

[38] Hustedt F. (1931). Diatomeen aus dem Feforvatn in Norwegen. Arch. Hydrobiol..

[39] Lange-Bertalot H., Moser G. (1994). *Brachysira*. Monographie der Gattung und *Naviculadicta* nov. gen. Bibl. Diatomol..

[40] Lange-Bertalot H., Genkal S.I. (1999). Diatoms from Siberia I. Islands in the Arctic Ocean (Yugorsky-Shar Strait). Iconogr. Diatomol..

[41] Genkal S.I., Jarushina M.I. (2017). *Sellaphora vekhovii* and *S. elorantana* (Bacillariophyta): Morphology, taxonomy, distribution in Russia. Nov. Sist. Nizshikh Rastenii.

[42] Carter J.R., Bailey-Watts A.E. (1981). A taxonomic study of diatoms from standing freshwaters in Shetland. Nova Hedwig..

[43] Genkal S.I., Yarushina M.I. (2016). A study of flora of Bacillariophyta in water bodies and water courses of the Naduiyakha River Basin (Yamal Peninsula, Russia). Int. J. Algae.

[44] Levkov Z., Nakov T., Metzeltin D. (2006). New species and combination from the genus *Sellaphora* Mereschkowsky from Macedonia. Diatom Res..

[45] Levkov Z., Krstic S., Metzeltin D., Nakov T. (2007). Diatoms of Lakes Prespa and Ohrid: About 500 taxa from ancient lake system. Iconogr. Diatomol..

[46] Metzeltin D., Lange-Bertalot H. (1998). Tropical diatoms of South America I: About 700 predominantly rarely known or new taxa representative of the neotropical flora. Iconogr. Diatomol..

[47] Glushchenko A.M., Kuznetsova I.V., Kulikovskiy M.S. (2021). The Diatoms of Southeast Asia.

